# JAK inhibition ameliorates bone destruction by simultaneously targeting mature osteoclasts and their precursors

**DOI:** 10.1186/s41232-023-00268-4

**Published:** 2023-03-03

**Authors:** Shinya Yari, Junichi Kikuta, Hotaka Shigyo, Yu Miyamoto, Daisuke Okuzaki, Yuki Furusawa, Masafumi Minoshima, Kazuya Kikuchi, Masaru Ishii

**Affiliations:** 1 Department of Immunology and Cell Biology, Graduate School of Medicine and Frontier Biosciences, Osaka University, Osaka, 565-0871 Japan; 2 WPI-Immunology Frontier Research Center, Osaka University, Osaka, Japan; 3 Laboratory of Bioimaging and Drug Discovery, National Institutes of Biomedical Innovation, Health and Nutrition, Osaka, Japan; 4 Genome Information Research Center, Research Institute for Microbial Diseases, Osaka University, Osaka, Japan; 5 AbbVie GK, Medical Affairs, Tokyo, Japan; 6 Department of Applied Chemistry, Graduate School of Engineering, Osaka University, Osaka, Japan

**Keywords:** JAK inhibitor, Osteoclast, Intravital imaging, Inflammatory bone destruction, Cell migration, Chemokine

## Abstract

**Background:**

Rheumatoid arthritis (RA) is characterized by chronic inflammation and resultant cartilage/bone destruction because of aberrantly activated osteoclasts. Recently, novel treatments with several Janus kinase (JAK) inhibitors have been shown to successfully ameliorate arthritis-related inflammation and bone erosion, although their mechanisms of action for limiting bone destruction remain unclear. Here, we examined the effects of a JAK inhibitor on mature osteoclasts and their precursors by intravital multiphoton imaging.

**Methods:**

Inflammatory bone destruction was induced by local injection of lipopolysaccharides into transgenic mice carrying reporters for mature osteoclasts or their precursors. Mice were treated with the JAK inhibitor, ABT-317, which selectively inhibits the activation of JAK1, and then subjected to intravital imaging with multiphoton microscopy. We also used RNA sequencing (RNA-Seq) analysis to investigate the molecular mechanism underlying the effects of the JAK inhibitor on osteoclasts.

**Results:**

The JAK inhibitor, ABT-317, suppressed bone resorption by blocking the function of mature osteoclasts and by targeting the migratory behaviors of osteoclast precursors to the bone surface. Further exhaustive RNA-Seq analysis demonstrated that *Ccr1* expression on osteoclast precursors was suppressed in the JAK inhibitor-treated mice; the CCR1 antagonist, J-113863, altered the migratory behaviors of osteoclast precursors, which led to the inhibition of bone destruction under inflammatory conditions.

**Conclusions:**

This is the first study to determine the pharmacological actions by which a JAK inhibitor blocks bone destruction under inflammatory conditions; this inhibition is beneficial because of its dual effects on both mature osteoclasts and immature osteoclast precursors.

**Supplementary Information:**

The online version contains supplementary material available at 10.1186/s41232-023-00268-4.

## Background

Rheumatoid arthritis (RA) is an autoimmune disease that results in severe bone destruction because of inflammation [1]. RA is characterized by synovial inflammation, hyperplasia of the joints, and destruction of both cartilage and bone. Bone erosion occurs rapidly from the onset of RA; it results in deformation and functional deterioration of the joints [2, 3]. Therefore, the treatment of RA involves suppression of progressive bone destruction.

Osteoclasts are multinucleated giant cells that differentiate from cells of the monocyte/macrophage lineage after stimulation with macrophage colony-stimulating factor and receptor activator of NF-κB ligand (RANKL) [4, 5]. Osteoclasts constitute a specialized subset of cells that resorb bone via acid secretion; they have a critical role in normal bone homeostasis [5]. However, inflammation-activated osteoclasts cause severe bone erosion and focal bone loss, which lead to joint destruction [6]. Previously, we developed an intravital multiphoton imaging system to visualize bone tissues in living mice; we have demonstrated the dynamic behaviors of osteoclasts and their precursors. We first visualized the migration and localization of osteoclast precursor macrophages that are finely controlled by a blood-enriched mediator of lipid metabolism, sphingosine-1-phosphate (S1P), representing a novel mechanism for the control of osteoclastogenesis and bone resorption in vivo [7, 8]. Furthermore, we visualized bone destruction by intact mature osteoclasts, which exhibit two distinct functional states: bone-resorbing (R) cells that are firmly attached to bones and actively dissolve the bone matrix and non-resorbing (N) cells that attach loosely to bones with less capacity for bone resorption [9, 10].

A range of agents have been developed and clinically applied for the treatment of RA and related arthritic diseases. Notably, biological disease-modifying antirheumatic drugs (bDMARDs), such as anti-tumor necrosis factor-alpha (TNF-α) antibody, anti-interleukin (IL)-6 receptor antibody, and cytotoxic T-lymphocyte antigen (CTLA)-4 Ig, are effective for limiting arthritic inflammation and bone destruction in patients [11, 12]. Using our intravital bone imaging system, we have directly visualized the cellular dynamics of mature osteoclasts and their precursors during inflammatory bone destruction; we revealed that different biologics acted at specific points in the process of osteoclastic bone destruction with varying efficacies. In particular, blockade of IL-6 receptor and TNF-α markedly inhibited the bone resorptive function of mature osteoclasts, whereas CTLA-4 Ig mainly affected the mobility of osteoclast precursors [13].

Janus kinase (JAK) inhibitors are emerging as a new class of agents for the treatment of RA [14–19]. JAK inhibitors, which exhibit effectiveness comparable to bDMARDs, can be administered orally because they constitute low-molecular-weight compounds. The JAK-signal transducers and activation of transcription (STAT) signaling pathway is a representative intracellular transduction pathway that is involved in many crucial biological processes, including cell proliferation and differentiation. Pro-inflammatory cytokines bind to their receptors on the cell membrane; the intracellular JAKs bound to the receptors are phosphorylated. The transcription factor STAT is activated by these JAKs; it regulates the gene expression patterns of several inflammatory molecules [20]. JAK is the collective term for a family of four tyrosine kinases consisting of JAK1, JAK2, JAK3, and tyrosine kinase 2 (TYK2). Specific proinflammatory cytokines activate particular members of the JAK family via signaling [21]. JAK inhibitors block activation of the JAK-STAT signal transduction pathway, leading to significant suppression of the progressive bone and articular destruction that occurs in RA. However, it remains unclear how JAK inhibitors affect osteoclast dynamics and functions, resulting in the suppression of bone erosion in vivo.

Using our intravital bone imaging system, we visualized the cellular dynamics of mature osteoclasts and their precursors. Specifically, we explored how the JAK inhibitor, ABT-317, which selectively inhibits activation of JAK1, affected osteoclast dynamics in vivo (e.g., the migration of osteoclast precursors and the activation of mature osteoclasts). We found that the JAK inhibitor blocked both the bone resorptive function of mature osteoclasts and the migration of osteoclast precursors to the bone surface during inflammatory bone destruction.

## Methods

### Mice

Wild-type C57BL/6 J mice were purchased from CLEA Japan. CX3C receptor 1-enhanced green fluorescent protein (CX_3_CR1-EGFP) knock-in mice were obtained from the Jackson Laboratory [22]. Tartrate-resistant acid phosphatase-tandem Tomato (TRAP-tdTomato) transgenic mice have been described previously [10]. All mice were maintained under a 12-h/12-h light/dark cycle in the specific pathogen-free animal facilities of Osaka University. All animal experiments were approved by the Institutional Animal Experimental Committee of Osaka University.

### Treatment with drugs

To perform intravital imaging, lipopolysaccharide (LPS) (10 mg/kg body weight; Sigma-Aldrich) dissolved in phosphate-buffered saline (PBS) was injected into the calvarial periosteum of mice under isoflurane anesthesia. To perform RNA sequencing (RNA-Seq), reverse transcription-quantitative polymerase chain reaction (RT-qPCR), and flow cytometry analyses, LPS (20 mg/kg body weight) dissolved in PBS was injected into the calvarial periosteum. From the day of LPS injection, the JAK inhibitor ABT-317, which was provided by AbbVie Bioresearch Center, at a dose of 60 mg/kg body weight dissolved in 0.5% hydroxypropyl methylcellulose (Alfa Aeser) and 0.02% Tween 80 (Sigma-Aldrich) in sterile water was orally administered twice daily for 5 days. To visualize osteoclastic bone resorption using intravital bone imaging, a pH-activatable fluorescent probe for osteoclast activity sensing (pHocas)-3 dissolved in PBS was subcutaneously injected at a dose of 6 mg/kg body weight daily into TRAP-tdTomato mice, beginning 3 days before imaging. The C–C motif chemokine receptor 1 (CCR1) antagonist, J-113863 (MedChemExpress, #HY-103360) (3 mg/kg body weight) dissolved in 10% dimethyl sulfoxide (Sigma-Aldrich), 40% polyethylene glycol 300 (Wako), 5% Tween 80 (Sigma-Aldrich), and 45% PBS, was injected intraperitoneally once daily for 5 days.

### Intravital multiphoton bone tissue imaging

Calvarial bone tissues of TRAP-tdTomato and CX_3_CR1-EGFP mice (7–20 weeks old) were examined by intravital multiphoton microscopy using a previously reported protocol with modifications [7–10]. The imaging system consisted of a Carl Zeiss upright multiphoton microscope (LSM 780 NLO) equipped with a 20 × water immersion objective (W Plan-Apochromat: numerical aperture (N.A.), 1.0; Carl Zeiss) and a Nikon upright multiphoton microscope (A1R MP +) equipped with a 25 × water immersion objective (CFI175 Apo 25XC W 1300: N.A., 1.1; Nikon). The imaging system for Carl Zeiss upright multiphoton microscopy was driven by a femtosecond-pulsed infrared laser (Chameleon Vision II Ti: Sapphire; Coherent, Inc.). The imaging system for Nikon upright multiphoton microscopy was driven by a dual femtosecond-pulsed infrared laser (Chameleon Discovery; Coherent, Inc.). Intravital bone imaging experiments in the absence of pHocas-3 were performed using a Nikon multiphoton microscope, whereas experiments in the presence of pHocas-3 were performed using a Zeiss multiphoton microscope.

Using a Carl Zeiss upright microscope, spectral imaging was performed with specialized internal multi-photomultiplier detectors. Acquired raw images were subjected to spectral unmixing with ZEN software (Carl Zeiss) to create unmixed images that excluded autofluorescence. An excitation wavelength of 940 nm was used to simultaneously excite second harmonic generation (SHG), autofluorescence, pHocas-3, and tdTomato. To obtain snapshot images, image stacks were collected in vertical steps of 3 µm at a depth of 50–150 µm below the skull bone surface with × 1.5 zoom, 512 × 512 X–Y resolution, and a time resolution of 5 min. Spectral unmixing was performed on intravital bone imaging data using a Zeiss multiphoton microscope. Fluorescence spectra of SHG, autofluorescence, pHocas-3, and tdTomato were obtained using ZEN software by manual selection of appropriate pixels on true color intravital bone images of wild-type mice, wild-type mice-administered pHocas-3, and TRAP-tdTomato mice, respectively. These spectral libraries were initially saved on a computer and used to create unmixed images via spectral unmixing algorithms in which each type of fluorescence was discriminated and autofluorescence was excluded [23].

Using a Nikon multiphoton microscope, multifluorescence images were acquired by direct detection of fluorescence using four external non-descanned detectors equipped with dichroics and emission filters, including an infrared-cut filter (DM685), three dichroic mirrors (DM458, DM506, and DM605), and four emission filters (492/SP for the SHG image, 525/50 for EGFP, and 583/22 for tdTomato). The excitation wavelengths were 880 nm for SHG and EGFP and 1040 nm for tdTomato. To obtain snapshot images, image stacks were collected in vertical steps of 3 µm at a depth of 90 µm below the skull bone surface with × 1.0 zoom and 512 × 512 X–Y resolution. For intravital time-lapse bone imaging to analyze the mean tracking speed of EGFP^+^ cells, image stacks were collected in vertical steps of 3 µm at a depth of 6–9 µm below the skull bone surface with × 1.5 zoom, 512 × 512 X–Y resolution, and a time resolution of 30 s.

### Image analysis of bone resorbing index in mature osteoclasts

To assess the bone resorption index of mature osteoclasts after image acquisition, NIS-Elements software (Nikon) was used. First, constant *γ* corrections were applied to all images to enhance the signal-to-noise ratio: tdTomato, *γ* = 0.9; pHocas-3, *γ* = 2.5. Mature osteoclast areas were binarized using Otsu’s thresholding method and automatically extracted from the original maximum intensity projection images. The mean pHocas-3 fluorescence intensities in mature osteoclast areas (pHocas-3 signals) and outside such areas (pHocas-3 noise) were measured. The bone resorption index was calculated as the ratio of pHocas-3 signal to pHocas-3 noise (Supplementary Fig. S1A) [23].

### Image analysis to track morphological changes in mature osteoclasts

Cell shapes were recognized using the NIS-Elements software (Nikon). We defined three distinct areas: initially (*t* = 0) (A); in the final time frame (*t* = 5) (C); and overlapping between these two time frames (B). The cell deformation index was calculated as (A + C)/(A + B), representing the ratio of area change over 10 min divided by the area change during the previous time frame. The details have been described previously (Supplementary Fig. S1B) [9].

### Image analysis of tracking speed mean of osteoclast precursors

To correct the drift of visual fields, raw time-lapse imaging data were processed using NIS-Elements software (Nikon). EGFP^+^ cells were automatically identified using Imaris software (Bitplane). This tracking algorithm uses autoregressive motion, with max distance of 6 µm and gap size of 3.

### Image analysis of the bone adhesion area of osteoclast precursors

CX_3_CR1-EGFP/TRAP-tdTomato mice were used in this analysis. The surface tool of Imaris software (Bitplane) was used to perform automatic cell-surface segmentation of each area of SHG^+^ bones, tdTomato^+^ mature osteoclasts, and EGFP^+^ osteoclast precursors. The 3D surface objects of bones were manually adjusted to the 3D surface objects of mature osteoclasts tightly adhering to the bone surface. EGFP^+^ surface objects ≤ 50 µm^3^ in volume were not included in the analysis, because such groupings were unlikely to represent cells. The surface tool was then used to detect colocalized SHG and EGFP voxels, defined as the adhesion areas between bones and osteoclast precursors (Supplementary Fig. S2A). EGFP^+^ osteoclast precursor areas and adhesion areas were binarized using Otsu’s thresholding method and automatically extracted from the edited images by NIS-Elements software (Nikon). The ratio of the sum of osteoclast precursor areas to the sum of adhesion areas was calculated.

### Flow cytometry and cell sorting

For sample collection, LPS was injected into the calvarial periosteum of CX_3_CR1-EGFP mice, together with JAK inhibitor or vehicle twice per day; 5 days later, bone marrow cells were collected from calvarial bone tissues with flow cytometry buffer (FACS buffer) (1 × PBS, 4% heat-inactivated fetal bovine serum, and 2 mM ethylenediaminetetraacetic acid) using a mortar. EGFP^+^ cells were then isolated from the extract using an SH800 cell sorter (Sony). Isolated murine cells were blocked with anti-CD16/32 antibody (BD Biosciences, #553141) for 15 min, followed by staining with anti-CD191 (CCR1)-phycoerythrin (BioLegend, #152507) and phycoerythrin-conjugated Rat IgG2b, *κ* isotype control antibody (BioLegend, #400607) for 30 min. Measurements such as the mean fluorescence index were performed using FlowJo software (TreeStar).

### RNA-Seq analysis

Cells were digested in QIAZOL (Qiagen) to extract total RNA, in accordance with the manufacturer’s instructions. The library was prepared using a TruSeq Stranded mRNA Sample Prep Kit (Illumina), in accordance with the manufacturer’s instructions. Sequencing was performed on an Illumina HiSeq 2500 platform in 75-base single-end mode. Illumina Casava 1.8.2 software was used for base calling. Bioinformatics analysis was performed using Integrated Differential Expression and Pathway (iDEP) analysis software, version 94. Access to raw data concerning this study was submitted under Gene Expression Omnibus (GEO) accession number GSE193104.

### Cell culture

For immunoblot analysis, murine macrophage RAW 264.7 cells were cultured with 100 nM or 2 µM ABT-317 or with vehicle for 1 h and then stimulated with IL-6 (RSD) at a concentration of 10 ng/mL or 1 ng/mL for 5 min or 10 min. Cell lysates were then prepared and subjected to immunoblotting.

For RT-qPCR analysis, the RNeasy Micro Kit (Qiagen) was used to extract mRNA from RAW 264.7 cells cultured for 24 h with 100 nM ABT-317 or vehicle in the presence of IL-6 at a concentration of 10 ng/mL. RT-qPCR was then performed as described below.

### Immunoblot analysis

Cell lysates prepared in a radioimmunoprecipitation assay (RIPA) buffer (Thermo Scientific) containing a protease inhibitor (cOmplete™, Mini, EDTA-free, Roche) and a phosphatase inhibitor (PhosSTOP™, Roche) were subjected to immunoblot analysis using antibodies specific for STAT1 (Cell Signaling Technology, #9172), Phospho-STAT1 (Cell Signaling Technology, #7649), STAT3 (Cell Signaling Technology, #4904), and Phospho-STAT3 (Cell Signaling Technology, #9145).

### RT-qPCR

Cells were digested in QIAZOL (Qiagen) to extract total RNA, in accordance with the manufacturer’s instructions. Total RNA and cDNA of the cells from bone tissues were obtained using RNeasy Mini Kit (Qiagen) and Superscript III reverse transcriptase (Thermo Fisher Scientific), in accordance with the manufacturer’s instructions. Quantitative reverse transcription-polymerase chain reaction was performed for 50 cycles using a Thermal Cycler Dice Real-Time System TP800 (TaKaRa Bio) with TB Green Premix EX Taq (Tli RnaseH Plus) and Bulk (TaKaRa Bio). The relative levels of the mRNA transcripts of interest were calculated using the 2^ΔΔCt^ method. The relative levels were normalized to the housekeeping gene, *Gapdh*, and the specificities of the amplified products were confirmed from dissociation curves. The following specific primer pairs were used (forward and reverse, respectively): *Gapdh* (5′-ACCACAGTCCCATGCCATCAC-3′ and 5′-TCCACCACCCTGTTGCTGTA-3′) and *Ccr1* (5′-GTGTTCATCATTGGAGTGGTGG-3′ and 5′-GGTTGAACAGGTAGATGCTGGTC-3′).

### Statistical analysis

Data shown are representative of at least three independent experiments unless otherwise indicated. All data were analyzed using GraphPad Prism 6 (GraphPad Software, Inc.) and are presented as means + or  ± standard deviations (SDs) unless otherwise stated. Statistical analyses were performed using the unpaired two-tailed *t*-test or the Mann-Whitney *U*-test for between-group comparisons. Statistical analyses of comparisons among three groups were performed using the Kruskal-Wallis test with Dunnett’s post hoc test and analysis of variance (ANOVA) with the Tukey’s post hoc test. In all analyses, *P* < 0.05 was considered to indicate statistical significance.

## Results

### JAK inhibitor suppressed the function of inflammation-activated mature osteoclasts

**Fig. 1:**
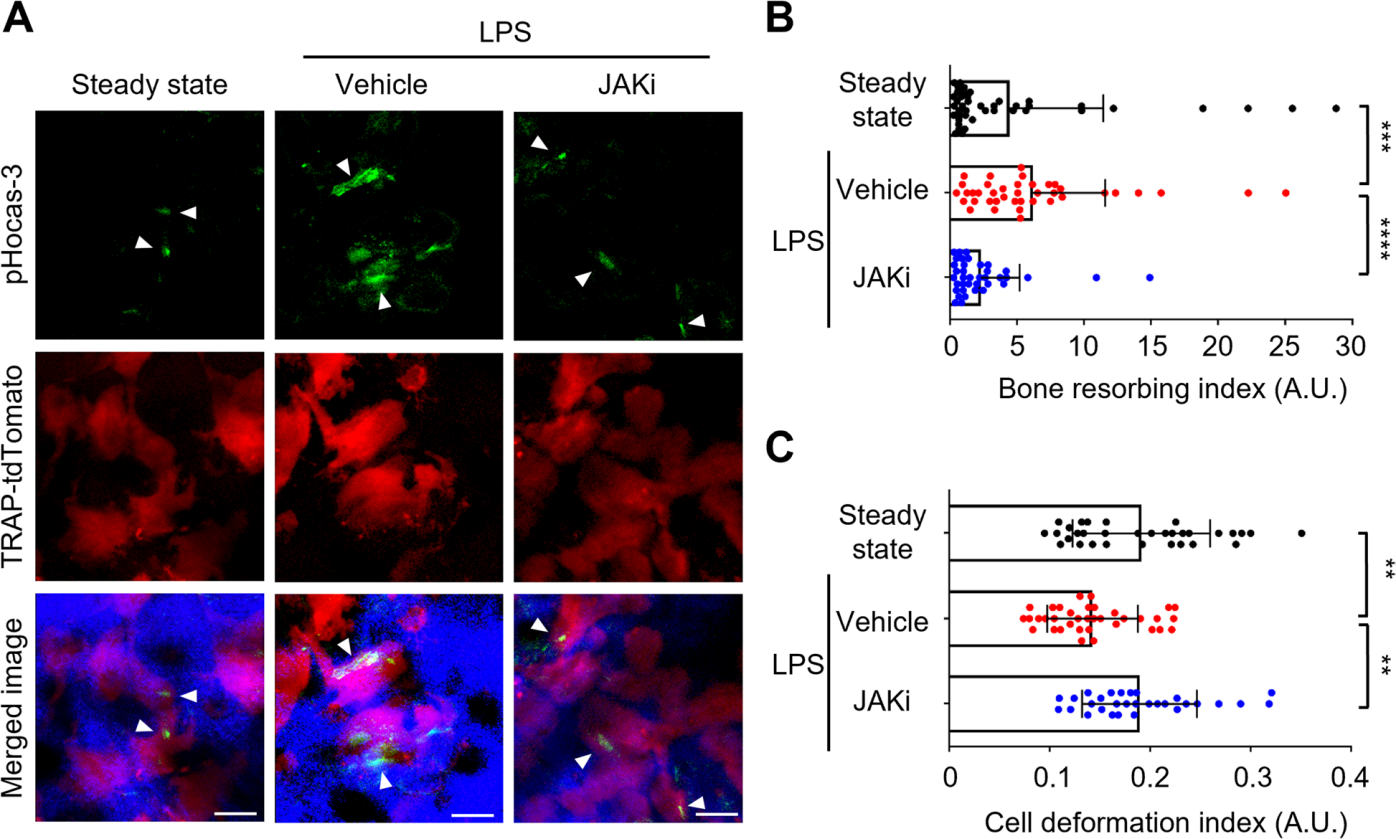
JAK inhibitor suppressed the function of inflammation-activated mature osteoclasts.** A** Representative intravital multiphoton imaging results of the bone resorptive activity of mature osteoclasts in calvarial bones in JAK inhibitor (JAKi)-treated LPS-induced mice. Green, pHocas-3; red, TRAP-tdTomato^+^ mature osteoclasts; blue, bones (second harmonic generation). Arrowheads: bone resorption areas. Scale bar, 30 µm. **B** The bone resorption index (BRI) of mature osteoclasts. A higher BRI indicates increased osteoclast activity (steady state, *n* = 43 images; vehicle, *n* = 40 images; JAKi, *n* = 38 images from 5 mice per group). Error bars indicate the means + SDs. **C** Mobility of mature osteoclasts. A higher cell deformation index (CDI) indicates increased mobility (steady state, *n* = 32 cells from 3 mice; vehicle, *n* = 36 cells from 3 mice; JAKi, *n* = 30 cells from 4 mice). Error bars indicate the means ± SDs. Statistical significance was determined by Kruskal-Wallis test. **P < 0.01, ***P < 0.001 ****P<0.0001

To investigate the effects of JAK inhibitor on the cellular dynamics of mature osteoclasts and their precursors in vivo, we used a lipopolysaccharide (LPS)-induced inflammatory bone destruction model, as described previously [13]. LPS causes bone destruction both directly and indirectly. In the former, LPS promotes osteoclast differentiation and activation through toll-like receptor 4 (TLR4) signaling [24, 25], and in the latter, LPS/TLR4 signaling induces the production of proinflammatory cytokines, which upregulate RANKL expression in osteoblasts or fibroblasts, leading to osteoclast activation [26, 27]. IL-6 is a proinflammatory cytokine that activates the JAK-STAT signaling cascade. As JAK inhibitors block JAK-STAT signaling, but not the LPS/TLR4 signaling pathway, they would inhibit osteoclast activation by suppressing IL-6-mediated RANKL production. First, we used intravital multiphoton microscopy to examine the effects of JAK inhibitor on the bone resorptive function of mature osteoclasts in living mice. To visualize osteoclastic bone resorption, we used mice expressing the reporter tdTomato in the cytosol of mature osteoclasts (TRAP-tdTomato mice) [9]; we also used pHocas-3, which is capable of detecting localized acidification by bone-resorbing mature osteoclasts on the bone surface in vivo [10]. LPS was injected into the calvarial periosteum of TRAP-tdTomato mice; the JAK inhibitor, ABT-317, was administered orally twice daily for 5 days. Five days after LPS injection, mouse skull bone tissues were visualized to assess the functions of mature osteoclasts (Fig. 1A, Supplementary Fig. S1A). Compared with steady-state conditions, the bone resorptive activity of mature osteoclasts was significantly activated under LPS-induced inflammatory conditions (Fig. 1 A and B, Supplementary Video 1). The bone resorptive activity was significantly inhibited in mice treated with JAK inhibitor, compared with vehicle-treated controls (Fig. 1 A and B, Supplementary Video 1).

Previously, we characterized two different populations of living mature osteoclasts in terms of their motility and function: static bone resorptive (R-type) and moving non-resorptive (N-type) cells [9]. To further investigate the effects of the JAK inhibitor on the dynamics of mature osteoclasts, we tracked morphological changes in mature osteoclasts (Supplementary Fig. S1B). Compared with mice in steady-state conditions, the motility of mature osteoclasts was significantly decreased in LPS-stimulated mice, indicating an increase in the population of R-type mature osteoclasts under inflammatory conditions (Fig. 1C, Supplementary Video 1). The motility of mature osteoclasts was significantly increased in mice treated with JAK inhibitor, compared with vehicle-treated controls (Fig. 1C, Supplementary Video 1), indicating an increase in the population of N-type mature osteoclasts. These results suggested that the JAK inhibitor affects the bone resorptive function of mature osteoclasts.

### JAK inhibitor influenced the migration of osteoclast precursors to the bone surface

**Fig. 2:**
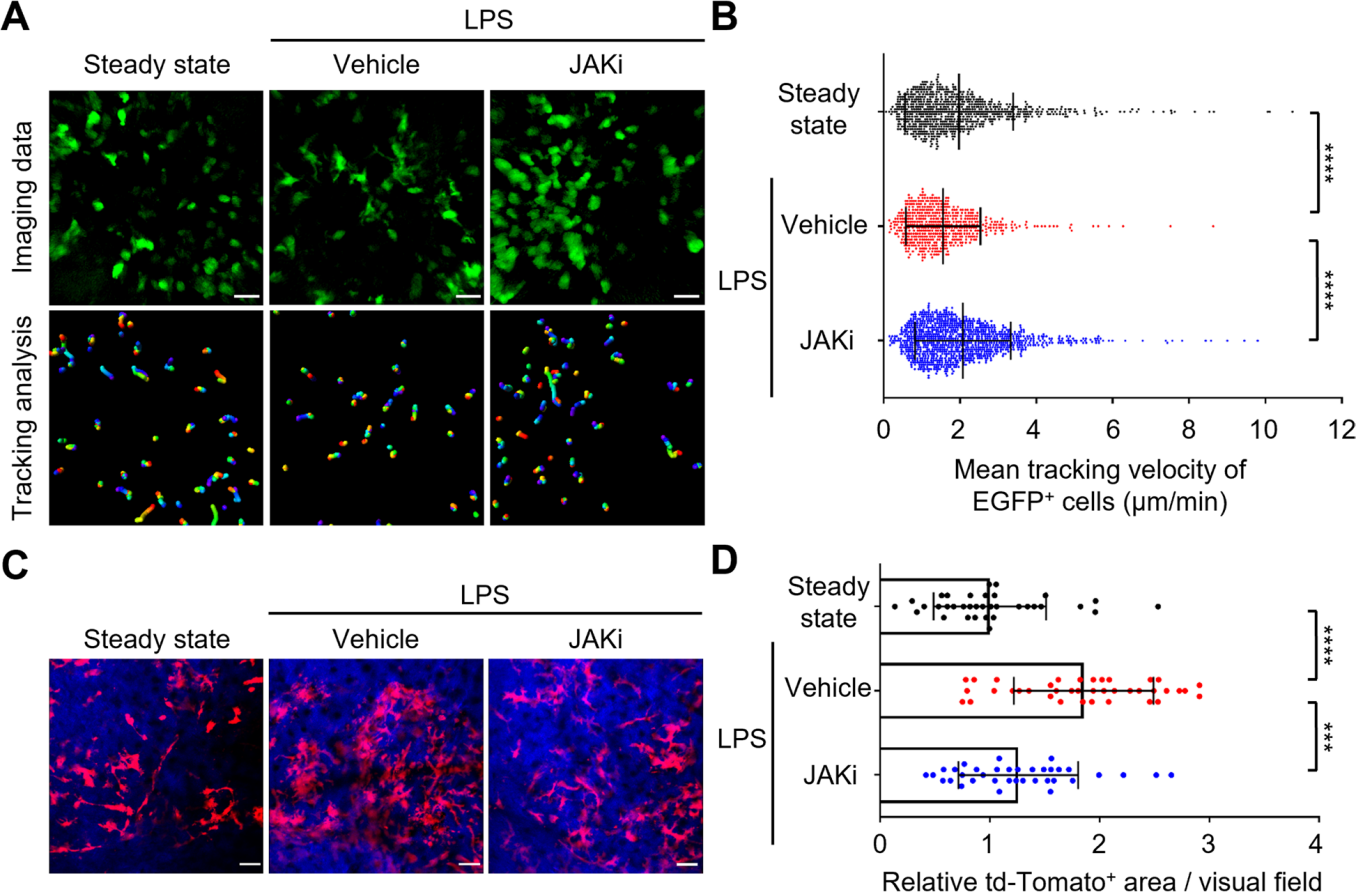
JAK inhibitor influenced migration of osteoclast precursors to the bone surface. **A** Representative intravital multiphoton imaging of the migration of osteoclast precursors in calvarial bones in JAK inhibitor (JAKi)-treated LPS-induced mice. Green, CX_3_CR1-EGFP. Scale bar, 30 µm (upper panels). The movement of CX_3_CR1-EGFP^+^ cells was tracked for 10 min. Colored lines show the cell trajectories (lower panels). **B** Tracking velocities of EGFP^+^ cells (steady state, *n* = 795 cells; vehicle, *n* = 570 cells; JAKi, *n* = 1091 cells from 5 mice per group). **C** Representative intravital multiphoton imaging of mature osteoclasts in calvarial bones in JAK inhibitor (JAKi)-treated LPS-induced mice. Red, TRAP-tdTomato^+^ mature osteoclast; blue, bones (second harmonics generation). Scale bar, 50 µm. **D** Area of TRAP-tdTomato^+^ mature osteoclasts relative to steady state (steady state, *n* = 35 images; vehicle, *n* = 39 images; JAKi, *n* = 36 images from 5 mice per group). Error bars indicate the means ± SDs. Statistical significance was determined by Kruskal-Wallis test. ***P<0.001, ****P<0.0001

Next, we focused on the effects of the JAK inhibitor on osteoclast precursors, which are the source of mature osteoclasts. LPS was injected into the calvarial periosteum of reporter mice expressing EGFP under the control of the CX_3_CR1 promoter to label osteoclast precursor monocytes (CX_3_CR1-EGFP mice) [7, 28, 29]. Five days after LPS injection, intravital multiphoton microscopy was used for visualization of mouse skull bone tissues to assess the mobility of EGFP^+^ monocytoid cells. The mean tracking velocity of CX_3_CR1-EGFP^+^ osteoclast precursors was significantly lower under LPS-induced inflammatory conditions than in steady-state conditions (Fig. 2 A and B, Supplementary Video 2). The osteoclast precursors tightly adhered to the endosteum in the inflammatory environment, which is regarded as a critical step during differentiation into mature osteoclasts [5]. In contrast, treatment with JAK inhibitor significantly increased the mean tracking velocity of osteoclast precursors compared with vehicle-treated controls (Fig. 2 A and B, Supplementary Video 2).

To clarify whether the JAK inhibitor interferes with the migration of osteoclast precursors to the bone surface, we analyzed the adherence of osteoclast precursors to the bone surface. The bones were visualized using the SHG signal; the adhesion area between CX_3_CR1-EGFP^+^ osteoclast precursors and bone was extracted from the original images (Supplementary Fig. S2A). Compared with steady-state conditions, the adhesion area was increased under LPS-induced inflammatory conditions (Supplementary Fig. S2 B, C). In contrast, the adhesion area was significantly smaller in JAK inhibitor-treated mice than in vehicle-treated controls (Supplementary Fig. S2 B, C), indicating that the JAK inhibitor prevented the migration of osteoclast precursors to the bone surface under inflammatory conditions associated with bone destruction.

Furthermore, to examine the number of mature osteoclasts at the site of LPS-induced inflammation, the areas of TRAP-tdTomato^+^ mature osteoclasts were automatically extracted from the original images and statistically evaluated (Fig. 2 C, D). In comparison with steady-state conditions, the TRAP-tdTomato^+^ area was significantly increased under LPS-induced inflammatory conditions. Treatment with JAK inhibitor decreased the area of mature osteoclasts (Fig. 2 C, D), compared with vehicle-treated controls. These results suggest that the JAK inhibitor has an inhibitory effect on the firm attachment of osteoclast precursors to the bone surface, thus limiting the number of mature osteoclasts.

### JAK inhibitor decreased the expression of *Ccr1* on osteoclast precursors under inflammatory conditions

**Fig. 3:**
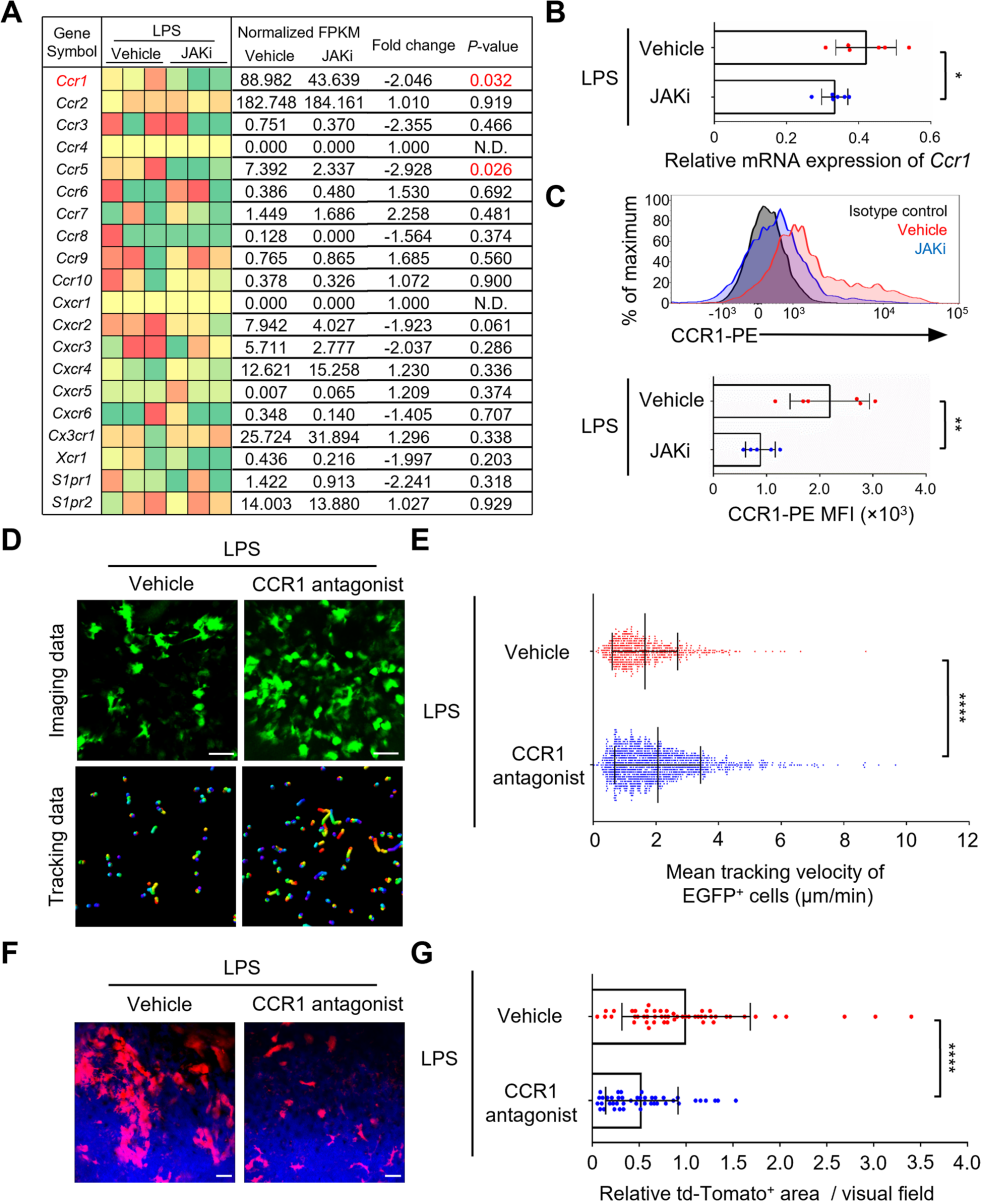
JAK inhibitor decreased expression of *Ccr1* in osteoclast precursors under inflammatory conditions. **A** Heatmap of representative receptors on CX_3_CR1-EGFP^+^ cells in JAK inhibitor (JAKi)-treated LPS-induced mice. Green, downregulation; red, upregulation (*n* = 3 mice per group). **B** Relative mRNA expression of *Ccr1* in CX_3_CR1-EGFP^+^ cells (*n* = 6 mice per group). **C** Representative histograms (upper) and quantitative mean fluorescence intensity (MFI) (lower) of CCR1 in CX_3_CR1-EGFP^+^ cells (vehicle, *n* = 6; JAKi, *n* = 5 mice). **D** Representative intravital imaging of osteoclast precursors of CCR1 antagonist-treated LPS-induced mice. Green, CX_3_CR1-EGFP^+^ cells. Scale bar, 30 µm (upper panels). Colored lines show the cell trajectories for 10 min (lower panels). **E** Mean tracking velocities of CX_3_CR1-EGFP^+^ cells (vehicle, *n* = 589 cells; CCR1 antagonist, *n* = 1281 cells from 6 mice per group). **F** Representative intravital imaging of mature osteoclasts. Red, TRAP-tdTomato^+^ cells; blue, bones. Scale bar, 50 µm. **G** Areas of TRAP-tdTomato^+^ mature osteoclasts relative to vehicle controls (vehicle, *n* = 51 images; CCR1 antagonist, *n* = 44 images from 6 mice per group). Statistical significance was determined by two-tailed Student’s *t*-test (**B**, **C**) and Mann-Whitney test (**E**, **G**). Error bars indicate the means ± SDs. *P < 0.05, **P < 0.01, ****P<0.0001

To determine the molecular mechanism by which the JAK inhibitor prevented the migration of osteoclast precursors to the bone surface, CX_3_CR1-EGFP^+^ cells were isolated from the calvaria of LPS-induced inflammatory bone destruction model mice treated with JAK inhibitor or vehicle; RNA sequencing (RNA-Seq) analysis was then performed. Several genes associated with osteoclast differentiation were downregulated upon treatment with JAK inhibitor (*Ocstamp*, *Dcstamp*, *Ctsk*, and *Atp6v0d2*) (Supplementary Fig. S3A). Gene Ontology (GO) pathway enrichment analysis showed that most of the downregulated genes were related to the regulation of cell migration (Supplementary Fig. S3B). Next, we examined changes in the gene expression patterns of representative chemokine receptors and S1P receptors (*S1pr1*, *S1pr2*) [7, 30], which are known to influence the mobility of osteoclast precursors in vivo (Fig. 3A). The predicted target gene affected by the JAK inhibitor was *Ccr1* (< − twofold change, *P* < 0.05). This downregulation was validated at both mRNA and protein levels (Fig. 3 B, C).

CCR1 is a representative inflammatory chemokine receptor expressed on monocytes, macrophages, immature dendritic cells, and natural killer cells [31] and is involved in the chemotaxis of osteoclast precursors in vitro [32]. According to the ChIP-Atlas, an integrative, comprehensive database [33], *Ccr1* expression is controlled by STAT1. In murine macrophage RAW 264.7 cells, its expression is increased by IL-6-induced STAT3 activation [34]. Whether the JAK inhibitors directly affect osteoclast precursors, by suppressing STAT phosphorylation and regulating *Ccr1* expression, was investigated in vitro using RAW 264.7 cells cultured with JAK inhibitor or vehicle for 1 h and then stimulated with IL-6 for 5 min or 10 min. STAT phosphorylation was then analyzed by immunoblotting. The results showed that the JAK inhibitor blocked the IL-6-induced phosphorylation of STAT1 and STAT3 (Supplementary Fig. S4 A and B). To examine the direct effect of the JAK inhibitor on *Ccr1* expression in osteoclast precursor, RAW 264.7 cells were cultured for 24 h with JAK inhibitor or vehicle in the presence of IL-6. The RT-qPCR analysis showed that the JAK inhibitor suppressed the IL-6-induced upregulation of *Ccr1* expression (Supplementary Fig. S4C). These results suggest that the JAK inhibitor acts directly on osteoclast precursors and decreases *Ccr1* expression.

Finally, we investigated whether CCR1 has an effect on the migration of osteoclast precursors to the bone surface under inflammatory conditions in vivo. LPS was injected into the calvarial periosteum of CX_3_CR1-EGFP mice; the CCR1 antagonist, J-113863, was administered intraperitoneally once daily for 5 days. Five days after LPS injection, intravital multiphoton microscopy was used to visualize the mobility of CX_3_CR1-EGFP^+^ osteoclast precursors in calvarial bone tissues (Fig. 3D). The mean tracking velocity of CX_3_CR1-EGFP^+^ osteoclast precursors was significantly faster in the CCR1 antagonist-treated mice than in vehicle-treated controls (Fig. 3 D and E, Supplementary Video 3). To clarify whether the CCR1 antagonist interferes with the migration of osteoclast precursors to the bone surface, we analyzed the adherence of osteoclast precursors to the bone surface. Compared with vehicle-treated controls, the adhesion area was decreased under CCR1 antagonist-treated mice (Supplementary Fig. S5 A, B). We also examined the effects of CCR1 antagonist on the number of mature osteoclasts at the site of LPS-induced inflammation using TRAP-tdTomato mice. Compared with vehicle-treated controls, the area of TRAP-tdTomato^+^ mature osteoclasts was significantly decreased in mice treated with CCR1 antagonist (Fig. 3 F, G). These results suggested that the JAK inhibitor decreases the expression of *Ccr1* on osteoclast precursors, resulting in the inhibition of their migration to the bone surface and a decrease in the number of mature osteoclasts.

## Discussion

**Fig. 4:**
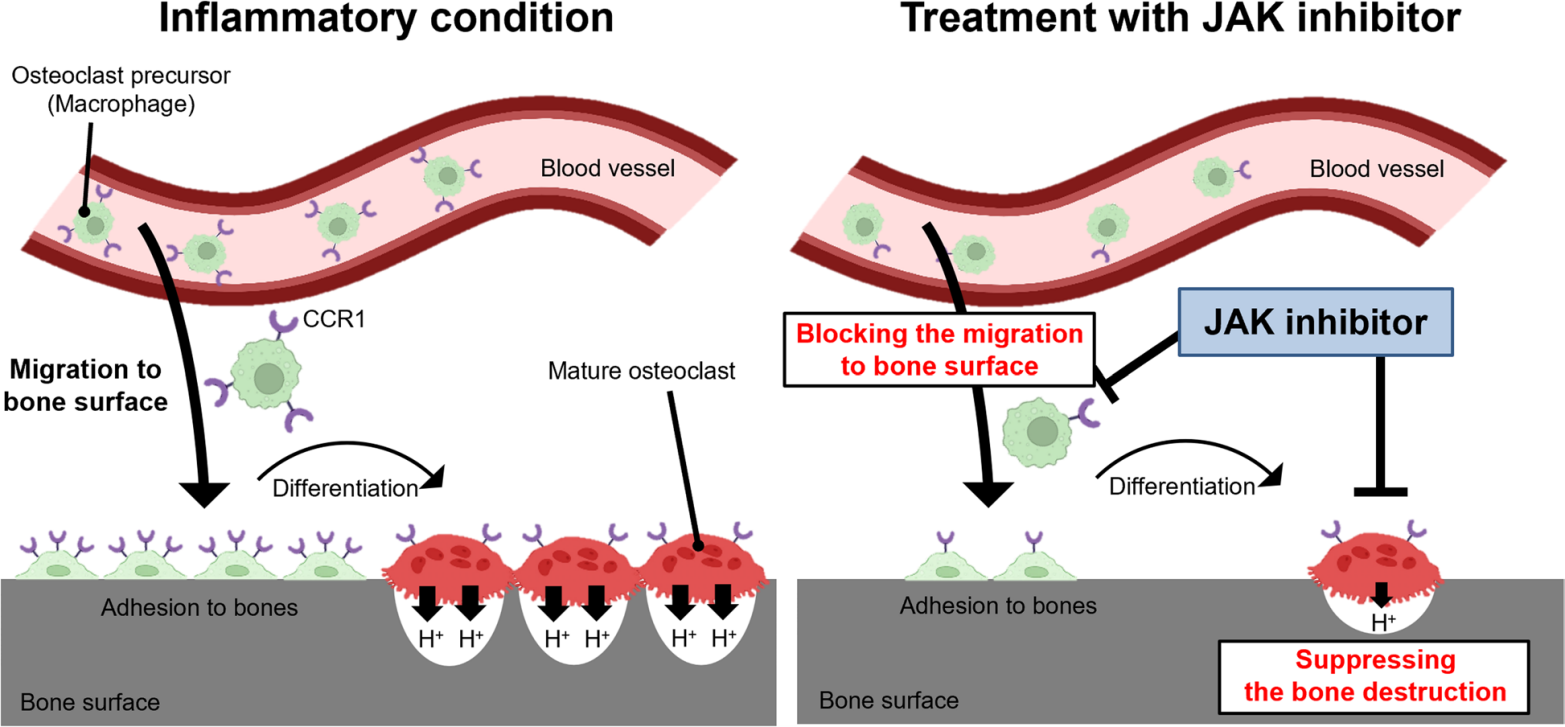
Scheme of the effects of JAK inhibitor on osteoclasts in vivo. Under inflammatory conditions, many osteoclast precursors migrate to the bone surface at the inflammatory site through CCR1-induced signaling. These cells differentiate into mature osteoclasts and cause abnormal bone destruction (left). JAK inhibitor decreased the expression of *Ccr1* on osteoclast precursors and blocked the migration of these cells to the bone surface, resulting in a decrease in the number of mature osteoclasts. In addition, JAK inhibitor suppressed the bone resorptive activity of mature osteoclasts (right)

JAK inhibitors are remarkable therapeutic agents to inhibit progression of structural joint damage in patients with RA [14–19]. In this study, we analyzed the in vivo effects of the JAK inhibitor, ABT-317, on the dynamics of mature osteoclasts and their precursors in a model of inflammatory bone destruction using intravital multiphoton microscopy. The results showed that the JAK inhibitor suppressed both the bone resorptive activity of mature osteoclasts and the migration of osteoclast precursors to the bone surface under conditions associated with inflammatory bone destruction (Fig. 4). In addition, we found that treatment with JAK inhibitor decreased the expression of *Ccr1* on osteoclast precursors. Imaging experiments with the CCR1 antagonist, J-113863, showed that CCR1 has a regulatory role in the migration of osteoclast precursors to the bone surface under inflammatory conditions. Taken together, these observations indicated that the JAK inhibitor regulated the migration of osteoclast precursors by suppressing the expression of *Ccr1*, resulting in a reduced number of mature osteoclasts on the bone surface (Fig. 4). Previously, we reported that anti-IL-6 receptor antibody and anti-TNF-α antibody mainly affected mature osteoclasts, whereas CTLA-4 Ig exclusively affected their precursors [13]. In contrast to these bDMARDs, the JAK inhibitor affected both mature osteoclasts and their precursors under conditions associated with inflammatory bone destruction, which should facilitate stricter control of bone destruction (Fig. 4).

The results of this study showed that the JAK inhibitor suppressed bone resorption by mature osteoclasts. There are several possible mechanisms for this inhibitory effect. A previous study showed that the plasma and hind paw tissue levels of inflammatory cytokines, such as IL-6, were decreased by treatment with JAK inhibitor in a rat model of adjuvant-induced arthritis [35]. JAK inhibitors may suppress inflammation-related IL-6 signaling; it may also abrogate osteoclastic bone resorption, which is abnormally activated by inflammatory cytokines. Another possible molecular mechanism is the downregulation of *Ccr5* in mature osteoclasts. In this study, RNA-Seq analysis showed that treatment with JAK inhibitor prevented the expression of *Ccr5* on osteoclast precursors under inflammatory conditions (Fig. 3A). In a previous study, *Ccr5*-deficient mature osteoclasts showed decreased bone resorption activity and increased cell deformation index in comparison with controls [36]. These results were consistent with the effects of JAK inhibitors on the in vivo dynamics of mature osteoclasts observed in the present study (Fig. 1 B, C). Further studies concerning a method for the isolation of mature osteoclasts from bone tissues are needed to identify the target molecules of JAK inhibitors on mature osteoclasts.

The results of this study showed that the JAK inhibitor blocked the migration of osteoclast precursors to the bone surface and decreased the number of mature osteoclasts. In addition, the JAK inhibitor appeared to prevent osteoclast differentiation because the expression levels of osteoclast-related genes (e.g., *Ocstamp* and *Dcstamp*) were decreased in osteoclast precursors from JAK inhibitor-treated mice (Supplementary Fig. S3A). A previous study showed that the JAK inhibitor, tofacitinib, did not regulate RANKL-induced osteoclast differentiation [35], but in another study, tofacitinib was shown to inhibit the IL-6-mediated activation of osteoclast differentiation in vitro [37]. When IL-6 binds to its receptor, JAK1 is activated and phosphorylated [20]. Thus, the selective JAK1 inhibitor used in this study, ABT-317, may have suppressed the IL-6-mediated activation of osteoclast differentiation under inflammatory conditions. Moreover, bone marrow cells of *Ccr1*-deficient mice reportedly have a low capacity of osteoclastogenesis, indicating that CCR1 plays an essential role in osteoclast differentiation [38]. The CD45^+^ CD11b^+^ CD115^+^ cell population with osteoclast differentiation potential is also decreased in bone marrow cells of CCR1 knockout mice [38]. In addition to suppressing osteoclast precursor migration by downregulation of *Ccr1*, JAK inhibitor may change the cell population of osteoclast precursors in bone marrow, thereby reducing the number of mature osteoclasts.

Previously, we reported that CTLA-4 Ig increased the mobility of osteoclast precursors and suppressed inflammatory bone destruction [13]. We performed RNA-Seq analysis of osteoclast precursors in LPS-induced inflammatory bone destruction model mice, which were treated with CTLA-4 Ig [13]. The RNA-Seq data showed that there were no changes in the expression patterns of chemokine receptors, such as *Ccr1* or *Ccr5* (Supplementary Fig. S6), suggesting that the molecular mechanisms underlying the inhibition of osteoclast precursor migration may differ between the JAK inhibitor and CTLA-4 Ig.

## Conclusion

In this study, intravital bone imaging was used to visualize the in vivo behavior of mature osteoclasts and their precursors under conditions associated with inflammatory bone destruction. The ability of ABT-317 to act on both mature osteoclasts and their precursors during osteoclastic bone destruction demonstrated the potential of this JAK inhibitor to limit bone destruction in arthritic regions. Future studies should examine pharmacological variance among the different JAK inhibitors used in the clinical setting. The information provided by our intravital bone imaging system may contribute to optimizing drug regimens.

## Supplementary Information

**Additional file 1: SupplementaryVideo 1.**

**Additional file 2: SupplementaryVideo 2.**

**Additional file 3: SupplementaryVideo 3.**

**Additional file 4: Fig S1. **Evaluation of osteoclastic function by intravital imaging. **Fig S2.** JAK inhibitor reduced the bone adhesion area of osteoclast precursors at the site of inflammation. **Fig S3.** RNA sequence-based transcriptional profiling of osteoclast precursors in JAK inhibitor-treated mice. **Fig S4.** JAK inhibitor acted directly on osteoclast precursors and decreased *Ccr1* expression. **Fig S5.** CCR1 antagonist reduced the bone adhesion area of osteoclast precursors at the site of inflammation. **Fig S6.** RNA sequence-based transcriptional profiling of osteoclast precursors in CTLA-4 Ig-treated mice.

## Data Availability

The authors confirm that the data supporting the findings of this study are available within the article or its supplementary materials. Raw data were generated at Osaka University. Access to raw data concerning this study was submitted under Gene Expression Omnibus (GEO) accession number GSE193104. Derived data supporting the findings of this study are available from the corresponding author M. I. and J. K. on request.

## Abbreviations

bDMARDs: Biological disease-modifying antirheumatic drugs
CTLA: Cytotoxic T-lymphocyte antigen
IL-6: Interleukin-6
JAK: Janus kinase
LPS: Lipopolysaccharide
PBS: Phosphate-buffered saline
pHocas: pH-activatable fluorescent probe for osteoclast activity sensing
RA: Rheumatoid arthritis
RANKL: Receptor activator of nuclear factor-kappa B ligand
RNA-seq: RNA sequencing
SHG: Second harmonic generation
STAT: Signal transducers and activation of transcription
TNF-α: Tumor necrosis factor-alpha
TLR4: Toll-like receptor 4
TRAP: Tartrate-resistant acid phosphatase
TYK2: Tyrosine kinase 2
