# Supplementary material for: JAK inhibition ameliorates bone destruction by simultaneously targeting mature osteoclasts and their precursors
Source: Inflamm Regen. 2023 Mar 3;43:18. doi: 10.1186/s41232-023-00268-4 (PMC9983229; doi:10.1186/s41232-023-00268-4)
Supplement: Supplementary file 4 — Additional file 4: Fig S1. Evaluation of osteoclastic function by intravital imaging. Fig S2. JAK inhibitor reduced the bone adhesion area of osteoclast precursors at the site of inflammation. Fig S3. RNA sequence-based transcriptional profiling of osteoclast precursors in JAK inhibitor-treated mice. Fig S4. JAK inhibitor acted directly on osteoclast precursors and decreased Ccr1 expression. Fig S5. CCR1 antagonist reduced the bone adhesion area of osteoclast precursors at the site of inflammation. Fig S6. RNA sequence-based transcriptional profiling of osteoclast precursors in CTLA-4 Ig-treated mice. [file 41232_2023_268_MOESM4_ESM.pdf]

Supplementary Information

Supplementary Figures

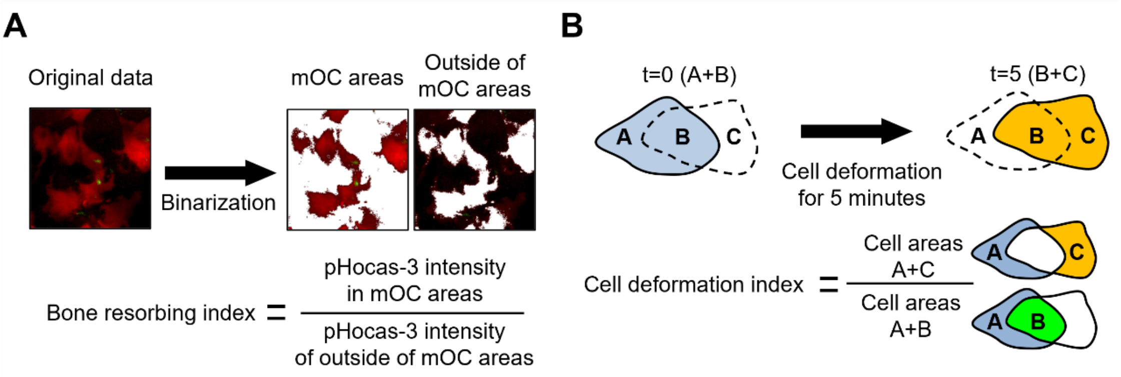

Supplementary Figure S1. Evaluation of osteoclastic function by intravital imaging. (A) Assessment of the bone resorptive activity of mature osteoclasts. Areas containing mature osteoclasts were automatically binarized from the original images. The mean pHocas-3 fluorescence intensities were measured inside (pHocas-3 signal) and outside the mature osteoclast areas (pHocas-3 noise). The bone resorption index was calculated as the ratio of pHocas-3 signal to pHocas-3 noise. Green, pHocas-3; red, TRAP-tdTomato$^+$ mature osteoclasts. (B) Cell shapes were automatically recognized by the image analysis software, and three distinct areas were defined initially (t = 0, A, cyan), in the final frame (t = 5, C, orange), and overlapping between these two frames (B, green). The cell deformation index was calculated as (A+C)/(A+B), representing the ratio of area change over 10 min divided by the area change during the previous time frame.

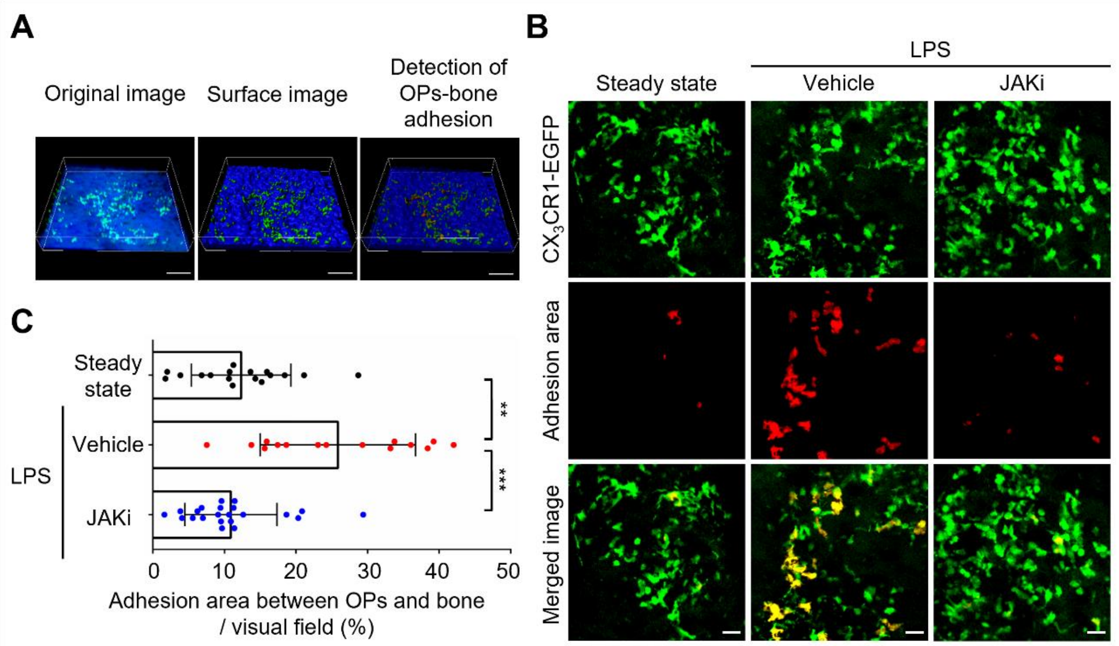

Supplementary Figure S2. JAK inhibitor reduced the bone adhesion area of osteoclast precursors at the site of inflammation. (A) Three-dimensional (3D) osteoclast precursor–bone adhesion analysis. A representative intravital multiphoton microscopic 3D image of the skull bone tissues. Green, osteoclast precursors expressing CX_3CR1-EGFP; blue, bone tissue (second harmonic generation) (left, original image). 3D surface rendering of osteoclast precursors and bone tissues from original image (middle, surface image). The contact area was defined as the adhesion area of osteoclast precursors and bone tissues (shown in red) (right, detection of osteoclast precursor–bone adhesion). Scale bar: 100 µm. (B) Representative osteoclast precursor-bone adhesion analysis. Upper panels show CX_3CR1-EGFP$^+$ cells. Middle panels show the adhesion area of CX_3CR1-EGFP$^+$ cells and bone tissues. Lower panels show merged images. Green, CX_3CR1-EGFP; red, contact area of EGFP$^+$ cell and bone tissue (second harmonic generation). Scale bar: 20 µm. (C) Adhesion area between osteoclast precursors and bone/visual field. Steady-state: $n = 17$ images from 3 mice;

vehicle: n = 15 images from 3 mice; JAK inhibitor (JAKi): n = 22 images from 4 mice. Statistical significance was determined by Kruskal-Wallis test. Error bars indicate the means ± SDs. **: $P < 0.01$, ***: $P < 0.001$.

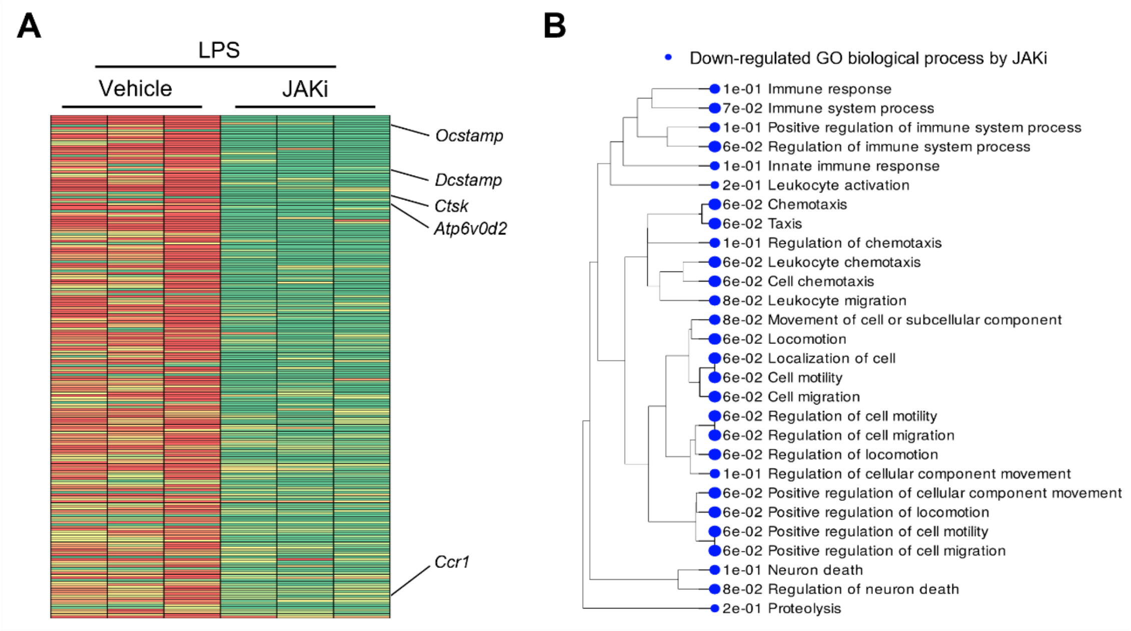

Supplementary Figure S3. RNA sequence-based transcriptional profiling of osteoclast precursors in JAK inhibitor-treated mice. (A) Heatmap of the genes downregulated by JAK inhibitor (JAKi) treatment. ≤ −2-fold change ($P < 0.05$; $n = 3$ mice per group). (B) Enriched Gene Ontology (GO) categories of differentially expressed genes between JAKi-treated LPS-induced inflammatory bone destruction model mice and vehicle-treated controls. Dot size is proportional to adjusted P-value.

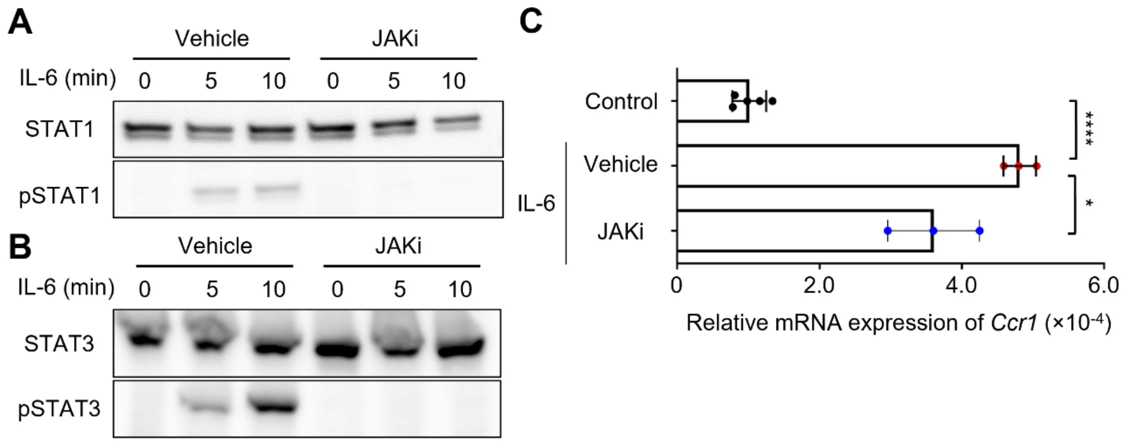

Supplementary Figure S4. JAK inhibitor acted directly on osteoclast precursors and decreased *Ccr1* expression. (A) RAW 264.7 cells pretreated with 100 nM JAK inhibitor (JAKi) or vehicle for 1 h were stimulated with IL-6 at a concentration of 10 ng/mL for 5 min or 10 min. Cell extracts were prepared and subjected to immunoblot analysis to detect the level of STAT1 phosphorylation (pSTAT1). (B) RAW 264.7 cells pretreated with 2 μM JAKi or vehicle for 1 h were stimulated with IL-6 at a concentration of 1.0 ng/mL for 5 min or 10 min. Cell extracts were prepared and subjected to immunoblot analysis to detect the level of STAT3 phosphorylation (pSTAT3). (C) Relative mRNA expression of *Ccr1* in RAW 264.7 cells. The cells were cultured for 24 h with 100 nM JAKi or vehicle in the presence of IL-6 at a concentration of 10 ng/mL. Control: n = 5 experiments; vehicle: n = 3 experiments; JAKi: n = 3 experiments. Statistical significance was determined in one-way ANOVA. Error bars indicate the means ± SDs. *: $P < 0.05$, ****: $P < 0.0001$.

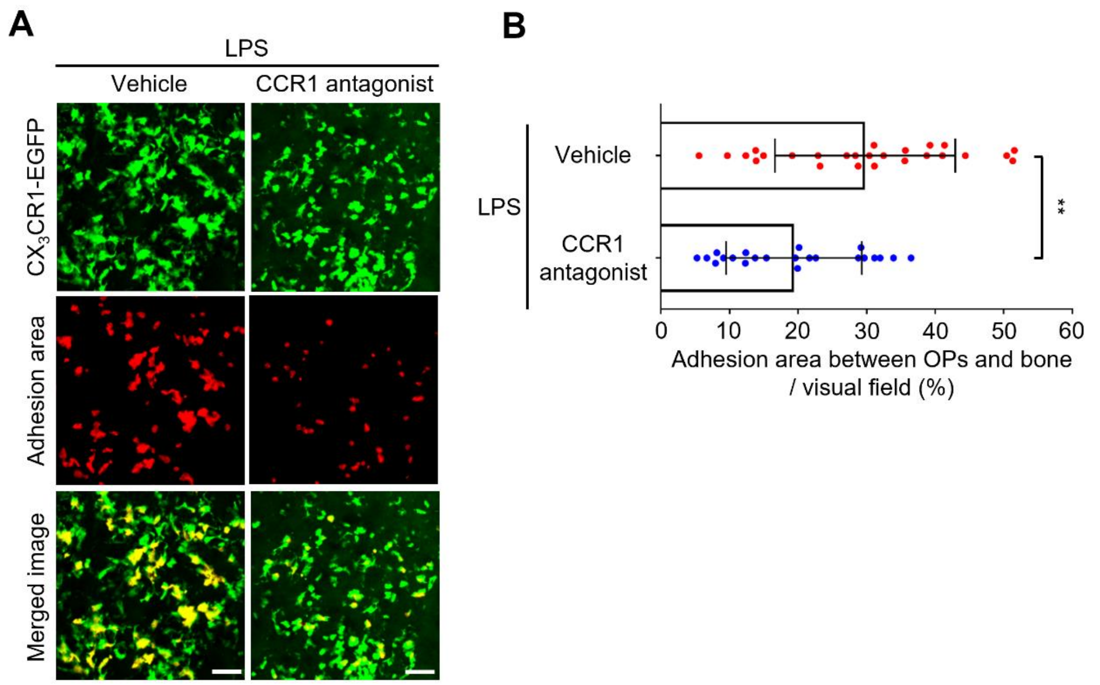

Supplementary Figure S5. CCR1 antagonist reduced the bone adhesion area of osteoclast precursors at the site of inflammation. (A) Representative osteoclast precursor-bone adhesion analysis. Upper panels show CX_3CR1-EGFP$^+$ cells. Middle panels show the adhesion area of CX_3CR1-EGFP$^+$ cells and bone tissues. Lower panels show the merged images. Green, CX_3CR 1-EGFP; red, contact area of EGFP$^+$ cell and bone tissue (second harmonic generation). Scale bar: 20 μm. (B) Adhesion area between osteoclast precursors and bone/visual field. Vehicle: $n = 26$ images from 3 mice; CCR1 antagonist: $n = 22$ images from 3 mice. Statistical significance was determined in Mann–Whitney U test. Error bars indicate the means ± SDs. **: $P < 0.01$.

Gene Symbol	LPS		Normalized FPKM		Fold change	*P*-value
	Vehicle	CTLA-4 Ig	Vehicle	CTLA-4 Ig		
Ccr1			88.114	86.333	-1.025	0.936
Ccr2			394.646	335.909	-1.173	0.130
Ccr3			0.886	0.757	-1.167	0.110
Ccr4			0.039	0.048	1.000	N.D.
Ccr5			8.469	7.440	-1.130	0.303
Ccr6			0.236	0.178	-1.222	0.766
Ccr7			0.565	0.831	1.668	0.256
Ccr8			0.037	0.000	-1.035	0.374
Ccr9			2.589	2.216	-1.073	0.853
Ccr10			0.659	0.739	1.104	0.773
Cxcr1			0.038	0.107	1.035	0.617
Cxcr2			29.605	41.764	1.466	0.182
Cxcr3			2.532	2.346	-1.059	0.727
Cxcr4			245.870	255.022	1.036	0.589
Cxcr5			0.141	0.057	-1.399	0.220
Cxcr6			0.630	0.734	1.223	0.526
Cx3cr1			22.862	22.416	1.038	0.913
S1pr1			0.748	1.311	1.670	0.184
S1pr2			10.836	11.526	1.069	0.686

Supplementary Figure S6. RNA sequence-based transcriptional profiling of osteoclast precursors in CTLA-4 Ig-treated mice. Heatmap of representative chemokine receptors and S1P receptors on CX_3CR1-$EGFP^+$ osteoclast precursors in CTLA-4 Ig-treated LPS-induced inflammatory bone destruction model mice and vehicle-treated controls. Green, downregulation; red, upregulation. The normalized fragments per kilobase of transcript per million mapped reads (FPKM) represents the mean FPKM for each group (n = 3 mice per group). Previously reported RNA-Seq data were modified [13].

Supplementary Videos

Supplementary Video 1. Intravital multiphoton imaging of TRAP-tdTomato$^+$ mature osteoclasts in skull bone of LPS-induced inflammatory model mice treated with JAK inhibitor. LPS was injected into the calvarial periosteum. Sequential images of the same visual field are shown. Green, pHocas-3. Red, TRAP-tdTomato. Scale bar: 40 μm.

Supplementary Video 2. Intravital multiphoton imaging of CX_3CR1-EGFP$^+$ cells in skull bone of LPS-induced inflammatory model mice treated with JAK inhibitor. LPS was injected into the calvarial periosteum. Sequential images of the same visual field are shown. Green, CX_3CR1-EGFP. Scale bar: 30 μm.

Supplementary Video 3. Intravital multiphoton imaging of CX_3CR1-EGFP$^+$ cells in skull bone of LPS-induced inflammatory model mice treated with CCR1 antagonist. LPS was injected into the calvarial periosteum. Sequential images of the same visual field are shown. Green, CX_3CR1-EGFP. Scale bar: 30 μm.
